# Revisional surgery in severe nutritional complications after bariatric surgical procedures: report of four cases from a single institution and review of the literature

**DOI:** 10.1590/0100-6991e-20202666

**Published:** 2021-01-13

**Authors:** JOÃO GABRIEL ROMERO BRAGA, MATHEUS MATHEDI CONCON, AMANDA PEREIRA LIMA, GUILHERME HOVERTER CALLEJAS, ARY DE CASTRO MACEDO, ELAINE CRISTINA CÂNDIDO, FELIPE DAVID MENDONÇA CHAIM, MURILLO PIMENTEL UTRINI, MARTINHO ANTÔNIO GESTIC, ALMINO CARDOSO RAMOS, EVERTON CAZZO, ELINTON ADAMI CHAIM

**Affiliations:** 1 - Universidade Estadual de Campinas, Departamento de Cirurgia Digestiva - Campinas - SP - Brasil

**Keywords:** Bariatric Surgery, Reoperation, Malnutrition, Cirurgia Bariátrica, Reoperação, Desnutrição

## Abstract

**Introduction:**

bariatric surgery is currently the only treatment that leads to long-term and sustained weight loss and decreased morbidity and mortality in morbidly obese individuals. Roux-en-Y bypass causes weight loss by restricting food intake associated with reduced intestinal absorption, in addition to multiple endocrine and satiogenic effects. Biliopancreatic diversion promotes weight loss mainly due to poor absorption of the nutrients ingested. Both procedures exclude parts of the gastrointestinal tract.

**Objective:**

to describe four cases of revisional surgery after primary bariatric surgery, due to serious nutritional complications, and to review the literature regarding this subject.

**Methods:**

a retrospective analysis of patients of Unicamps bariatric center database and review of the literatures were performed.

**Results:**

four patients were identified, 2 women and 2 men, with a mean age of 48 years. The mean body mass index before revisional surgery was 23.7 kg/m^2^. Three patients underwent Scopinaro biliopancreatic diversion, and onde patient underwent Roux-en-Y gastric bypass. The revisional surgeries were revision, conversion, and reversion. One patient died. For the review of the literature 12 articles remained (11 case reports and 1 case series). Another five important original articles were included.

**Conclusion:**

fortunately, revision surgery is rarely necessary, but when indicated it has increased morbidity, It can be revision, reverion or conversion according to the severity of the patient and the primary surgery performed.

## RESUMO

**Introdução:**

atualmente, a cirurgia bariátrica é o único tratamento que leva à perda de peso prolongada e sustentada e diminuição da morbimortalidade em indivíduos obesos mórbidos. O bypass em Y-de-Roux causa perda de peso restringindo a ingestão de alimentos associada à redução da absorção intestinal, além de múltiplos efeitos endócrinos e sacietógenos. O desvio biliopancreático promove a perda de peso principalmente devido à diminuição da absorção dos nutrientes ingeridos. Ambos os procedimentos excluem partes do trato gastrointestinal.

**Objetivo:**

descrever 4 casos de cirurgia revisional após cirurgia bariátrica primária, devido a graves complicações nutricionais, bem como realizar uma revisão da literatura sobre esse assunto.

**Métodos:**

foi realizada uma análise retrospectiva dos pacientes do banco de dados do serviço de cirurgia bariátrica da Unicamp e revisão da literatura.

**Resultados:**

foram identificados 4 pacientes, 2 mulheres e 2 homens, com média de idade de 48 anos, o IMC antes da cirurgia revisional eram em média 23,7. Os pacientes haviam sido submetidos em 3 casos a cirurgia de Scopinaro e 1 caso ao bypass gástrico em Y-de-Roux. As cirurgias revisionais foram de revisão, conversão e reversão. Um paciente evoluiu a óbito. Em relação a revisão da literatura, foram selecionados 12 artigos (11 relatos de casos e 1 série de casos). Outros cinco artigos originais importantes foram incluídos.

**Conclusão:**

felizmente, a cirurgia revisional raramente é necessária, mas, quando indicada, apresenta alta morbidade. São cirurgias de revisão, reversão ou conversão, de acordo com a gravidade do paciente e a cirurgia primária realizada.

## INTRODUCTION

In 2016, more than 1.9 billion adults, 18 years and older, were overweight. Of these over 650 million were obese^1^. Bariatric surgery is currently the only treatment that leads to long-term and sustained weight loss and decreased morbidity and mortality in morbidly obese individuals^2^. However, lifestyle modifications that include changes in diet and increased physical activity usually result in inefficient weight loss and inadequate maintenance of weight in the long-term^3^.

Roux-en-Y bypass causes weight loss by restricting food intake associated with reduced intestinal absorption, in addition to multiple endocrine and satiogenic effects. Biliopancreatic diversion (BPD) promotes weight loss mainly because of poor absorption of nutrients. Both procedures exclude parts of the gastrointestinal tract, which cause potential development of metabolic deficiencies and malabsorption of certain nutrients, including proteins and certain minerals and vitamins^4^.

Clinically relevant malabsorption should be considered if a patient shows one or more of the following symptoms/signs after the bariatric surgical procedure: gastrointestinal symptoms/signs, including diarrhea, abdominal distention, flatulence, abdominal pain and ascites, and other general symptoms, such as persistent weight loss, anemia, amenorrhea, impotence, infertility, night blindness, xerophthalmia, peripheral neuropathy, tiredness, fatigue, and weakness^5^. After the malabsorption procedure, a blood test panel must be requested, together with a regular postoperative follow-up, from the preoperative period, then in between the 3^rd^ and 6^th^ -month in the first two years, and annually thereafter^6^.

The present study aims to report the clinical aspects and treatment of four patients who underwent revisional surgery after primary bariatric surgery due to serious nutritional complications and to review the literature on this subject.

## METHODS

Based on a retrospective analysis of data collected from the medical records in Unicamp’s bariatric surgery center database, four cases of malnutrition revisional surgery were reported. The collected variables were age, sex, body mass index (BMI) before revisional surgery, bariatric surgery, and revisional surgery, follow-up before and after revisional surgery, outcomes, and reasons for revision.

Literature published in the last 10 years was reviewed through an online search for MeSH terms “Bariatric surgery” and “Malnutrition” in Medline (via PubMed) and Lilacs (via Bireme). Inclusion criteria were original studies, case reports, or case series of patients who underwent bariatric surgery, developed malnutrition or related conditions, and required revisional surgery. Articles reporting in vitro or animal studies, articles wherein participant characteristics did not meet the inclusion criteria, abstracts from poster presentations, review articles, and duplicate publications were excluded. Other articles were used for contextualization and discussion. Finally, four cases are presented from this bariatric center.

Based on a retrospective analysis of data collected from medical records, 4 cases of malnutrition revisional surgery reported in this bariatric center were added to the total number of cases reviewed in the literature over a 10-year period.

The study protocol was approved by the institutional research ethics board (reference number: Unicamp 4.018.799/CAAE: 30636620.3.0000.5404. 

## RESULTS

Four patients, 2 women and 2 men with a mean and standard deviation age of 48 ± 15.7 years, who underwent revisional surgery for severe nutritional complications were reviewed (Table 1). The mean and standard deviation BMI before bariatric surgery was 48± 3.49 kg/m^2^. The mean and standard deviation BMI before revisional surgery was 23.7± 2.86 kg/m^2^. The time between the first surgery and the revision surgery was 11 ± 2.34 years Regarding comorbidities, 2 patients did not have any, 1 had a depressive disorder and another had high blood pressure, hypothyroidism and depression disorder. 

**Table 1:** Main characteristics of the four patients who underwent revisional surgery for malnutrition.

Case	Age/ Sex	BMI (kg/m^2^) before revisional surgery	Bariatric surgery	Revisional surgery	Follow-up before revisional surgery	Follow-up after revisional surgery	Outcomes	Reasons for revision
Case 1	50 / M	26	Scopinaro BDP	Reversion	No	_	Enteric fistula	Liver failure
Case 2	71 / F	22	Scopinaro BDP	Reversion	No	20 months	Satisfactory	Malnutrition, hydroelectrolytic disorders, acute renal failure and anasarca
Case 3	27 / F	18	Roux-en-Y bypass	Reversion	No	24 months	Satisfactory	Progressive weight loss, Malnutrition and BMI of 18
Case 4	44 / M	23.1	Scopinaro BDP	Conversion	No	50 months	Satisfactory	Hypoalbuminemia, anemia, deficiencies of all fat-soluble vitamins and anasarca

BMI - body mass index; BDP - Biliopancreatic diversion.

Three patients underwent Scopinaro BPD^7^, and one patient underwent Roux-en-Y bypass. The revisional operations were conversion in one case, revision in another, and reversion in two patients.

All patients did not undergo regular postoperative follow-up and did not use the recommended supplementation. After revisional surgery, one patient died because of septic complications due to an enteric fistula and liver failure. He underwent 2 procedures to control the fistula, but he died 45 days after the initial surgical approach. The other 3 patients had regular follow-up, with a mean and standard deviation of 31.3 ± 13.2 months. 

For the review of the literature, the total number of articles by database search was 156 and 13 in PubMed and Lilacs, respectively. After excluding duplicates and screening by title and abstract, 12 articles remained (11 case reports and 1 case series). Another five important original articles were included. Eventually, 92 patients were found (Table 2), and a cumulative sample size of 96 patients from 9 different countries was evaluated. Of these patients, 15 died (16.3%). Patients underwent revision surgery for findings indicating severe malnutrition, such as anemia, hypoalbuminemia, anasarca, renal failure, and liver failure. The performed operations were characterized by revision procedures to elongation of the common limb; conversion operations, when a more disabsorptive procedure was converted into a less disabsorptive; and reversal operations, when a mechanism was used to establish normal anatomy. In this study, 11 (11.45%), 31 (32.29%), and 54 (56.25%) patients underwent conversion, reversion, and revision procedures, respectively.

**Table 2:** Reported cases of patients who underwent revisional surgery for malnutrition.

Author	N Bariatric/ N Revisional	N Revisional Surgery for Malnutrition	Age	BMI (kg/m^2^) Before	Bariatric Surgery	Revisional	Follow-up (months)	Outcomes	Revisional Cause
Willaert et al.	_	17	36.2 ± 8.3	45.5 ± 8.4	BPD (11) BPD-DS (4) RYGB (2)	Conversion (8) Revision (9)	28 ± 29	Satisfactory 14/17	Persistent diarrhea, BMI under 20 kg/m^2^, TPN
Pires Souto et al.	810/67	28	41.6 ± 10.4	51.4 ± 9.0	BDP-DS (25) JIB (2) RYGB (1)	Revision (25) Reversion (3)	19 ± 4.2	Satisfactory 58/67	_
Patel et al.	384/151	11	_	18 to 80	JIB (4) RYGB (4) MGB (1) VBG (2)	Reversion (9) Conversion (2)	_	Satisfactory 10/11	Severe chronic diarrhea, renal failure
Sampaio Neto et al.	_	2	_	38.61 and 51.92	RYGB (n=2)	Reversion (2)	_	Satisfactory 2/2	Severe undernutrition and electrolyte imbalance.
Caris F. et al.	1	1	43	32.2	RYGB (1)	Reversion (1)	4	Satisfactory 1/1	Anemia, Vitamin deficiency and severe malnutrition
Chousleb et al.	3726/259	6	51.2	_	RYGB (2) JIB (4)	Reversion (6)	1 to 36	Satisfactory 6/6	Anemia,renal failure, electrolyte imbalance
Spyropoulos et al.	1161/56	15	_	_	BPD-RYGBP (15)	Revision (15)	65 ± 7	Satisfactory 15/15	TPN and hypoalbuminemia
Tong et al.	1	1	58	53.2	BPD-DS (1)	Revision (1)	96	Satisfactory 1/1	Diarrhea that did not respond to dietary and medical therapy
Pitt et al.	1	1	38	42	Distal RYGB (1)	Revision (1)	48	Satisfactory 1/1	Kwashiorkor
Ceneviva et al.	1	1	55	60	RYGB (1)	Reversion (1)	36	Satisfactory 1/1	Steatorrhea, hypoalbuminemia, anemia
Motamedi et al.	1	1	37	44	MGB (1)	Reversion (1)	_	Satisfactory 0/1	Liver failure
Halawani et al.	1	1	37	49	BPD-DS (1)	Reversion (1)	6	Satisfactory 1/1	BMI under 20 kg/m^2^, hypoalbuminemia, deficiencies vitamins
Zubiaga et al.	1	1	47	48	MGB (1)	Revision (1)	3	Satisfactory 1/1	Diarrhea, steatorrhea, hypoalbuminemia and Wipple's disease
Appresai et al.	1	1	60	65	Distal RYGB (1)	Revision + Gastrostomy (1)	6	Satisfactory 1/1	Diarrhea, steatorrhea, hypoalbuminemia
Beghdadi et al.	1	1	36	50.1	Distal RYGB (1)	Reversion (1)	12	Satisfactory 1/1	Diarrhea, anasarca, hypoalbuminemia, neuropathy, liver failure
Martins et al.	1	1	47	52.9	RYGB (1)	Reversion (1)	18	Satisfactory 1/1	Anasarca, hypoalbuminemia, anemia, diarrhea
Akusoba et al.	_	3	56 ± 7.84	36.9 and 42.4 and 46.4	RYGB (1)	Reversion (3)	12	Satisfactory 3/3	BMI under 20kg/m^2^, hypoalbuminemia
Braga et al.	_	4	48 ± 15.7	48 ± 3.49	RYGB (1) BPD (3)	Conversion (1) Revision (1) Reversion (2)	31.3 ± 13.2	Satisfactory 3/4	Liver failure, acute renal failure and anasarca

NR - not reported - BMI: body mass index; BPD - biliopancreatic diversion; BPD-DS - biliopancreatic diversion with duodenal switch; RYGB - Roux-en-Y gastric bypass; JIB - jejunoileal bypass; MGB - mini-gastric bypass; VBG - vertical banded gastroplasty; TPN - total parenteral nutrition.

## DISCUSSION

Revisional bariatric operations are associated with higher complication rates than the initial procedures, regardless of the used technique, and according to the literature, rates vary from 13% to 55% in different studies^8^. Out of the 96 reviewed cases, 81 patients had successful outcomes, whereas 15 patients died, accounting for a mortality rate of 16.3%, which shows the complexity of a revisional operation for malnutrition.

Low adherence to follow-up has detrimental effects on patient safety due to the delay in diagnosing complications. Most bariatric surgery centers have standardized protocols for postoperative care; nevertheless, the literature reports that the follow-up of these patients can reach only 50% after 1 year of the operation^9^. 

Indications for revisional procedures are most often related to protein malabsorption, which is the most serious and potentially fatal side effect^10^. In addition to postoperative follow-up, monitoring these patients preoperatively is extremely important to identify signs of malnutrition. A study conducted in this bariatric center compared individuals who underwent a preoperative interdisciplinary bariatric surgery program with those who did not. The major outcomes included decreased length of hospital stay, wound dehiscence, wound infection, pulmonary complications, fistulas, pulmonary thromboembolism, sepsis, incisional hernias, reoperations, and mortality^11^.

With regard to the clinical presentation, patients with severe malnutrition requiring revisional operations, usually have hypoalbuminemia, anemia, renal failure, liver failure, ascites, anasarca, and they require preoperative parenteral nutrition^8^ ^,^ ^12^ ^-^ ^27^.

No randomized studies have evaluated the best revisional approach, and a consensus has not been reached to date. These procedures can be classified into revisions, conversions, and reversals. Revision procedures (elongation of the common channel) and conversion techniques are mostly associated with malabsorption, such as BPD, BPD with duodenal switch, and distal gastric bypass, into conventional gastric bypass are widely used techniques and they also maintain a metabolic component to prevent obesity relapse. Reversals are obviously chosen in patients with severe malnutrition or those who underwent revisional surgery^8^ ^,^ ^12^ ^-^ ^27^.

Several options can be considered, including proximal side-to-side anastomosis between the alimentary and biliopancreatic limbs or the alimentary limb that can be sectioned above the interstitial anastomosis as close as possible to the angle of Treitz^10^.

Akusoba et al.^27^ proposed an algorithm for managing patients with excessive weight loss. A gastrostomy must be performed in the excluded stomach for patients without strictures in the gastrointestinal tract, without psychiatric disorders, and with good caloric intake. However, a reversal procedure should be performed if the patient does not improve. A gastrostomy can be a good option for clinical and nutritional improvement, and precede the definitive treatment. Appresai and Murr^24^ described the use of gastrostomy with revision surgery as a way to nourish the patient in the postoperative period. The gastrostomy tube was removed eight weeks postoperatively.

Sampaio-Neto et al.^14^ described a revisional procedure and were successful in two cases. It consists of anastomosis between the alimentary limb 10 cm from the anterior gastroenterostomy and the gastric antrum 4 cm from the pylorus; the alimentary limb is sectioned distally to the new anastomosis, following the resection of the original enteroanastomosis. Ceneviva et al.^20^ described another type of revisional surgery, wherein the duodenum and proximal jejunum were reconnected to the alimentary tract. The alimentary limb was sectioned and stapled close to the jejunojejunal anastomosis and then anastomosed to the second part of the duodenum with a linear-stapled side-to side suture. Akusoba et al.^27^ described a reversion technique with transection of the gastric pouch proximal to the gastrojejunostomy, creation of a gastrogastrostomy, transection of the biliopancreatic limb off the jejunojejunostomy, and creation of an anastomosis between the biliopancreatic and Roux limbs transected from the gastrojejunostomy.

This study evaluated 96 patients (4 in our bariatric center and 92 in the literature review). Even after an extensive review, the descriptions of patients who were reoperated for malnutrition after bariatric surgery were scarce; hence, new publications, consensus, and clinical trials are necessary to define a follow-up model for these patients and the best surgical approach to be adopted.

## CONCLUSION

Severe malnutrition after bariatric surgery requiring surgical intervention is uncommon, but when present, it is associated with high mortality that is not negligible. Therefore, bariatric surgeons must be prepared to manage these difficult cases.

After bariatric surgery, patients should be assessed nutritionally on a regular basis. Malnutrition cases can be manifested in a drastic way, such as chronic diarrhea, acute renal failure, liver failure. Revision surgery is rarely necessary, but when indicated it is associated with increased morbidity. The revision procedure can be revision, reversion or conversion according to the severity of the patient and the primary performed operation.
